# Forms of life: a literary formalist view on biological individuality

**DOI:** 10.1007/s40656-025-00671-9

**Published:** 2025-05-14

**Authors:** Teun Joshua Brandt

**Affiliations:** 1 Department of European Literature and Culture, University of Groningen, Groningen, The Netherlands; 2 Department of Theoretical Philosophy, University of Groningen, Groningen, The Netherlands

**Keywords:** Biological individuality, Strategic formalism, Sociopolitical forms, New materialism, Interdisciplinary analysis

## Abstract

This article argues for a formalist approach to biological individuality, bridging formalist ways of reading in cultural and literary studies with contemporary debates in the philosophy of biology. Central to this discussion is the idea that the question of what constitutes an individual, spanning across domains such as biology, politics, law, and literature, is essentially a question of form: the conditions by which we individualise enforce a specific pattern through which we interpret the world, whether it is the natural world, the social world, or the fictional world of a literary text. Taking this as a starting point, the article adopts a strategic formalist method as articulated by Caroline Levine, employing a close-reading method that asks how forms of individuality, whether they are phenomenal, theoretical, or cultural, operate as they move beyond their designated system of discourse; what they afford when they travel across dissimilar materials; and what occurs when they intersect with other forms, be they sociopolitical, poetic, or aesthetic. Considering literary and sociopolitical forms on the same plane of existence as theoretical forms of individuality enables a needed conversation on the affordances of forms in both the production of knowledge and in the cultural imagination.

## Introduction

In narrative studies and literary theory, there is a common tendency to view the world of fictional narratives as populated by (fictional) individuals who, being the main actants of the story, drive the plot. The “fundamental compass of narrative is a world of individuals,” Félix Martínez-Bonati writes (1981, p. 24). Uri Margolin writes that for possible world semantics, “character is viewed as signifying an individual” (1987, p. 108). But what do we mean when talking about characters as being or signifying *individuals*? Surely, that they are one instead of many. Or that they are particulars instead of universals, which Ian Watt famously asserted to be the key aspect of the modern novel (Watt, 1957). But the term invokes more than that: individuals are commonly understood as distinct, indivisible entities, reflecting our intuitive understanding of our biological ‘selves’ and that of other organisms (Bouchard & Huneman, 2013; Elwick, 2017; Wilson, 1999). For twenty-first-century biology, however, the idea of the distinct, bounded organism has given way to more comprehensive approaches that embrace the abundant diversity of life-forms and prioritise complex modes of (co)existence (Gilbert et al., 2012; Suárez, 2018). While vastly different from each other in argument, most of these new theories share the same implication: the traditional understanding of individuality, based on the long-lasting idea of the anatomic, bounded organism, has not done justice to the complexities of life, and we should develop a conceptual space *beyond the bounded organism*. In light of this significant epistemic shift, we, as literary scholars, should begin to reconsider our conceptions of fictional individuals from the standpoint of biology and start asking questions that have yet to be answered: In regard to the literary imagination, by which criteria do we individualise, and how does this affect our reading*?* Do fictional narratives also reflect changes in our scientific understanding of life? When considering such questions of individuality, how do we navigate the interplay between the literary and the scientific?

With the intention of constructing a theoretical framework that will ultimately allow us to answer these questions, this article develops a formalist approach to individuality, inspired by the recent work of Caroline Levine. In literary studies, particularly concerning early formalist schools of thought such as Russian Formalism and Structuralism, ‘form’ typically denoted a work’s structural design, poetic patterns, and distinctive style, often juxtaposed with its content. Similarly, a traditional distinction exists between forms evident within a literary text and those shaping our sociopolitical reality. In her book *Forms: Whole, Rhythm, Hierarchy, Network,* Caroline Levine deconstructs this divide between form, content and context, and offers a theoretical framework for studying how literary forms influence thinking beyond the text and, vice versa, how sociopolitical forms shape literary texts, structuring aesthetic experience—a method that she names strategic formalism. What underscores her argument for employing such a method is that all of the uses of the term, despite the heterogeneity at the heart of its conceptual history, share a common definition: “‘form’ always indicates an arrangement of elements—an ordering, patterning, or shaping” (Levine, 2015, p. 3). Because forms are abstract organising principles, they are portable, “capable of imposing their arrangements on both social life and literary texts” (2015, p. 5). I argue that a strategic formalism is also productive for understanding the interrelation between the structuring and ordering of life in the biological sciences and literary forms, such as the character. This argument is grounded in the idea that the question of what constitutes an individual, spanning across domains such as biology, politics, law, and literature, is essentially a question of form: the conditions by which we individualise enforce a specific pattern through which we interpret the world, whether it is the natural world, the social world or the fictional world of a literary text.

Taking this as a starting point, Levine’s formalist approach offers a valuable lens for analysing and understanding the dynamics of forms of individuality within a broader cultural and social context. Contrary to much of the discussion on biological individuality in biology and the philosophy of biology, this approach does not seek to provide new insights into the epistemic value^1^ of biological individuality in general or the truth value of any particular form of individuality. Instead, it aims to contribute to the understanding of how forms of individuality, whether they are phenomenal, theoretical, or cultural, operate as they move beyond their designated system of discourse; what they *afford*—to speak with Levine’s proposed term—when they travel across dissimilar materials and what occurs when they *intersect* with other forms, be they sociopolitical, poetic, or aesthetic. Levine proposes a close reading strategy that pays “careful attention to the ways that historical texts, bodies, and institutions are organized—what shapes they take, what models they follow and rework” (2006, p. 632). My focus will be on the ordering of life-forms as individuals, in biology, but especially beyond—considering biological individuality “as the complex juncture of bio-social spaces,” as Alison McConwell aptly puts it (2023, p. 57). Informed by an increasing body of scholarship on the interrelation between form, narrative, and science (Beer, 2009; Duncan, 2019; G. Levine, 1992; Morgan et al., 2022; Newman, 2022a, b), I recognise the pivotal role of cultural forms at play in these bio-social spaces.

This article consists of four sections, structured to elucidate a formalist approach to biological individuality. In the first section, I outline the debate on biological individuality and introduce the notion of form within this context. The second section explores how these forms of individuality carry political power by shaping and anticipating social structures and relations. The third section outlines Levine’s formalist position and argues that a strategic formalism is productive for understanding the interrelation between the structuring and ordering of life in the biological sciences and the forms that structure works of culture as well as shaping our sociopolitical imageries—the shared narratives, metaphors and frameworks through which societies conceptualize political structures, social relations, and collective identities. I stress the relevance of narrative fiction for a formalist analysis of biological individuality—on the one hand, because it epitomizes the biopolitical operations of cultural forms, shaping normative or ideologically driven models of individuality such as the bounded organism; on the other, because narrative, in particular, serves as a site where sociopolitical, scientific, and literary forms collide, generating (potentially unforeseen) configurations, some of which may foster a biological imagination *beyond the bounded organism*, attuned to the various ways that life takes form, reflecting the broad scope of debate in biology and the philosophy of biology—a collision that, therefore, demands closer attention. In the final section, I examine Levine’s ideas in the context of New Materialist thought, considering the materiality of discourses and the semiotics of materials, with forms of individuality taking centre stage. Throughout the discussion, the bounded organism emerges as a travelling entity, playing a crucial role in both scientific and cultural imagination and bearing significant implications for the (de)construction of self, identity, and belonging.

## Forms of individuality

First, individuality is a form in itself, albeit a very basic one: it is what distinguishes one element from another and establishes its identity. In other words, individuality organises the living world into distinct elements, things, and processes. Biological individuals, in turn, are what we conceive of as living entities that form a ‘whole’ and can therefore, in some form or another, be delineated from their environment.^2^ “In general,” Thomas Pradeu writes, “asking what a biological individual is means asking what constitutes a countable, relatively well-delineated, and cohesive unit in the living world” (2016b, p. 762). Throughout much of biology’s history, the organism has been the prevailing paradigm for biological individuality (Wilson & Barker, 2024). An organism is a complex living system “comprised of diverse parts which work together to maintain the system’s structure, despite turnover of material, by making use of sources of energy and other resources from their environment” (Godfrey-Smith, 2013, p. 25). Thus, organismality is both an integrating and individuating form; what ‘emerges’ from the interplay of multiple parts are metabolically circumscribed entities that are relatively well-integrated and function as a whole. In other words, organisms are *whole systems* of interconnected and interdependent parts (cells, tissues, organs). However, there are also numerous life forms composed of multiple biological individuals who collectively form a ‘whole’ greater than the sum of their parts, yet they are not organisms in the traditional sense. Consider, for example, ‘superorganisms’ such as ant colonies, which exhibit behaviours and properties that suggest a kind of collective individuality. Other problems posed in this regard include rhizomatic plants (where does one end and the other begin?) or large fungal networks and swarms (is it one or multiple individuals?). While the organism has been a valuable model for individuality for most of biology’s history, its paradigmatic position has been constantly contested throughout the history of biology, defied by a plethora of anomalies from ‘normal science’, to speak in Kuhn’s terms.

The problem of circumscription—both in terms of (metabolic) boundedness and (genetic) distinctiveness—has become especially problematic in light of complex symbiotic entanglements. At first, symbiosis was considered an anomaly in itself: an exception to the overall struggle over resources that drives life’s evolution. However, since the early days of symbiosis research, the exception has become the rule, and we now know that the majority of life-forms are entangled in a myriad of intricate symbiotic relationships and that symbiosis also provides a common evolutionary mechanism (Gontier, 2015). Our immunity, metabolism and cognition are co-constituted by the vast majority of bacteria and viruses occupying the space that is our body (Bazin et al., 2022; Cox et al., 2022; Palacios-García & Parada, 2021). Such intricacies of interspecies relations blur the lines of boundaries between individuals: it is difficult to argue for being ‘metabolically circumscribed’ when a significant portion of metabolic turnover is outsourced to another organism. Moreover, through symbiosis, entities can integrate into other entities, much like Matryoshka dolls, ultimately forming a new, inseparable ‘emergent’ whole, necessitating a reconsideration of individuality throughout the process. Consider mitochondria in eukaryotic cells, which are now widely accepted to have evolved from free-living bacteria (Gray, 2012). These bacteria were engulfed by a host cell through a process called endosymbiosis, or symbiogenesis, and over time, this endosymbiotic relationship evolved to the point where the mitochondria became fully integrated into the eukaryotic cell, demonstrating how symbiosis can lead to the emergence of new forms of individuality at a different level of biological organization (Margulis, 1998). The horizontal gene transfer that accompanies processes such as endosymbiosis fundamentally challenges individuality based on (genetic) distinctiveness, resulting in multispecies genomes within complex multispecies bodies (Crisp et al., 2015; Reynoso-García et al., 2022). “The one-genome/one-organism doctrine of classical genetics has been eclipsed by studies of hereditary symbiosis,” Gilbert et al. write (2012).

To address these challenges, the concept of the holobiont has been proposed (Margulis & Fester, 1991; see also Simon et al., 2019), suggesting that an individual is not merely the host organism but rather a *composite* of the host and its associated symbiotic microorganisms. This concept acknowledges the interconnected nature of life forms and the crucial role that symbiotic relationships play in shaping their biology. Given that aspects such as health, immunity, and cognition—traditionally ascribed to the organism—are increasingly recognised as emergent properties of the interplay between organismal hosts and microbes, there is sufficient reason to use the holobiont as a valuable framework for scientific practice and theory (Kartjito et al., 2023; Palacios-García & Parada, 2021; Suárez & Triviño, 2020). Whether holobionts are units of selection, coevolutionary partnerships, or neither remains a topic of heated debate (cf. Zilber-Rosenberg & Rosenberg, 2008; Doolittle & Both, 2017; Godfrey-Smith, 2013; Douglas & Werren, 2016). Either way, holobionts challenge many established frameworks—being simultaneously multitudes of individuals and individuals of multitudes, functioning as both ecosystems and biological individuals (Suárez & Stencel, 2020).

A particular point of view is that of process-based ontology, for which individuals should be understood as stabilised processes instead of things (Nicholson & Dupré, 2018). Autopoietic living systems like organisms are good examples of this: they embody a process of dynamic stability, constantly keeping themselves far from equilibrium, rather than being static entities, despite our often perceiving and describing them as such. In this milieu, form is always in process, always precarious, and boundaries never fully realised. A process-based ontology also illuminates “why some scholars treat a holobiont as an organism and others do not. They are simply focusing on different temporal parts of the processes in question” (Stencel & Wloch-Salamon, 2022). This is why processual philosophers of biology also tend towards a pluralist stance, acknowledging multiple ways of drawing boundaries (Dupré, 1995; Stencel & Wloch-Salamon, 2022). A specific position within pluralism is promiscuous individualism, as articulated by John Dupré, which asserts that there is not only heuristic value but also ontological legitimacy to pluralism. Dupré summarises this view as follows: “there are various ways of drawing … boundaries, reflecting real biologically salient aspects of the multiply interconnected systems that make up the biological world” (2014, p. 241).

I view this broad diversity of models of biological individuality as representing a multitude of forms of individuality, each delineating the temporal and spatial organisation of life differently. Swarms, holobionts, organisms, composite organisms, communities, networks, clonal organisms, biofilms, superorganisms, etc.—they are forms of life in the sense of the term ‘life-form’, as characterised by Helmreich and Roosth, who argue that the term life-form points to “a space of possibility within which life might take shape” (2010, p. 27). The term has a “constitutive incompleteness” and is therefore deployable to new situations where former frameworks of terminologies lack (2010, p. 27). Rather than drawing a priori conclusions about the specific form of the object of study, it can refer to any entity that can be considered an object of study, whether it is a microbial mat, an Aspen grove, or a wild cat. Following both the definition by Helmreich and Roosth and the one by Levine, we can argue the following: whereas form in general relates to all spatial arrangements of elements, ‘life-form’ refers to the specific shapes and configurations in which life is thought to come into being. Forms of individuality, then, encompass a spectrum of such manifestations, each enforcing a specific pattern through which we interpret the world. They are ‘forms of order’, as Tobias Cheung puts it (2010). “It is the work of form to make order,” Levine resonates (2015, p. 3). But why adopt such a broad term as ‘form’ in this context? Why introduce another term that might obscure the conversation that philosophers of biology and biologists have constructively engaged in thus far?

## A biopolitics of individuality

First, since forms inherently create order, as Levine argues, they are ‘the stuff of politics’ (2015, p. 3). Similarly, “if the political is a matter of imposing and enforcing boundaries, temporal patterns, and hierarchies on experience, then there is no politics without form” (2015, p. 3). This is a crucial note to a debate that focuses primarily on epistemic questions. Levine’s interpretation of form as inherently political builds to a great extent on the work of Michel Foucault,^3^ who argued that political power is not just enacted by people or politics but, foremost, by the administrative structures that order bodies in society and that become embodied,^4^ continuing to circulate in society as systems of discipline. These range from temporal forms such as timetables to spatial forms like the panopticon (Foucault, 1977). This power of form is expressed in discourse and diffused through socialisation, but most of all, it determines what knowledge comes to be (Foucault, 1980). Of course, we can consider societal forms, such as the structures of research funding that determine which research agendas are included or excluded from knowledge production. A network can carry political power, as exemplified by the subversive qualities of blockchain systems. Alternatively, consider the way grassroots movements act through decentralised networks, evading the fragility of a top-down structure. However, on a different level, and much more pervasive in their subtlety, we can also consider the administrative structures that knowledge itself generates, amplifying or deconstructing biopolitical agendas. The influence of gender binarism on our everyday lives offers ongoing proof of this, as well as the enforced hierarchies between high/low, white/black, have/have not, etc.

Forms of individuality might be less obvious structures of biopower than gender binarism or hierarchical taxonomies, but the (sometimes intimate) relationship between theories of individuality and politics has been highlighted in numerous studies (Cheung, 2010; Clarke, 2020; Elwick, 2017; Gilbert, 2017; McConwell, 2023; Schneider, 2024; Trappes, 2024). As Alison K. McConwell points out, biological individuality has a dark side, “which serves as a cautionary tale wrapped in social and political ideologies about progress and perfection in nature … [it] has been used to promote politics of social ideologies about managing human evolution through use of the life sciences” (2023, p. 61). The eugenics promoted by early 20th-century Darwinists such as Julian Huxley built largely on narratives of individuality, portraying human organismal complexity as the pinnacle of evolution and its autonomy as justification for further advancing this progress. This narrative of progress is intricately linked to liberal valorisations of individuality, contrasted with fascist and communist ideologies of collectivity (McConwell, 2023, p. 78), highlighting the reciprocal influence between sociopolitical valorisations of individuality and biological theories. “For the political liberal,” Adam Ferner writes, “the human subject has to be single, complete and the ultimate proprietor of its own body and actions […] The discrete, boundaried self is an essential posit for those who need a ‘natural’ agent to prefigure (and thus stand as foundations) for market relations” (2020, p. 225). Angela Creager writes in a similar manner that “the atomistic and competitive view of the genetic individual that emerged fit well with Cold War political liberalism and (as others have noted) gave no evolutionary standing to symbiosis, cooperation, and other forms of biological collectivism” (2022, p. 251). “All of this is just to say,” as Adam Ferner puts it succinctly, “that there is distinct political pressure for us to conceive of ourselves as genuinely unified, rational beings with distinct, discrete, impermeable boundaries” (2020, p. 225). Central to Ferner’s categorisation of the modern liberal appreciation of the individual is the idea of autonomous agency, that is, in his words, being the ultimate proprietor of one’s own body and actions. In *Stories Americans Live By* (2005), Dan P. Adams argues in accordance, that in modern Western thought, individuals are seen as potentially self-sufficient agents with inherent rights, and society is composed of independent agents who consciously choose their actions. In *Sources of the Self* (1992), Charles Taylor argues that the modern concept of self is rooted in the idea of humans being autonomous individuals. With this in mind, it is hardly surprising that the concept of evolution driven by sheer competition among individuals for limited resources reached extreme levels in the liberal West of the early twentieth century, leaving little room for emerging research on symbiosis and its role in evolutionary novelty. When Lynn Margulis revived Konstantin Mereschkowski’s ridiculed theory of symbiogenesis, she faced intense criticism; her pioneering paper on endosymbiosis—arguing that organelles like mitochondria originated from symbiotic microbial relationships—was rejected by about 15 journals before its eventual acceptance (Knoll, 2012; Sapp et al., 2002). As Bruce Clarke puts it aptly: “Symbiosis was once a doubtful, even derided topic in biology, because its emphasis on ensembles and collectives of living beings ran counter to the larger discipline’s inheritance of Western and modern valorizations of individuality” (2020, p. 235). The history of biological individuality, thus, cannot be understood without recognising the political aspect of the debate. But how does this connect to formalist theory in literary and cultural studies?

## Strategic formalism

Through the course of literary studies’ formalist thought history, form was conventionally regarded as independent from its sociopolitical context, primarily carrying aesthetic value. However, at least since Frederic Jameson’s publication of *The Political Unconscious* (1981) and Terry Eagleton’s *Form and Content* (1976), there has been an increased focus on the relation between sociopolitical forms (of power) and literary forms, giving rise to a new wave of formalist criticism. Jameson advances an analytic attentiveness to the *ideology of forms*, as he calls it, that is, the “symbolic messages transmitted to us by the coexistence of various sign systems which are themselves traces or anticipations of modes of production” (1982, p. 62). Terry Eagleton argues in a similar vein that “form is the product of content, but reacts back upon it in a double-edged relationship” (1976, p. 20). As Levine points out in her 2006 article, for Marxist critics such as Jameson or Eagleton, “literary forms do not merely reflect social relationships but may help bring them into being” (2006, p. 625). They are not only traces but also *anticipations*, and to study literary forms means to examine the subtle ideological interventions of (textual) structures in our everyday interpretation of the world around us.

To offer a comprehensive method for studying the power of (literary) form in our everyday perception of the world, Levine extends the formalist awareness of Marxist critics such as Jameson and Eagleton by bringing together the many dispersed insights “into social and aesthetic forms to produce a new formalist method,” which she names *strategic formalism* (2015, p. 3). Levine’s book *Forms: Whole, Rhythm, Hierarchy, Network* (2015) should be understood as a first effort to construct such a method—with an open call for further development—based on five ideas that, until now, “have remained largely implicit … and disconnected from one another” (p. 4), namely, that forms *travel*, *constrain*, *differ*, *overlap and intersect*, and *do political work in particular historical contexts* (p. 6). What underscores these ideas is the understanding that, while forms of a literary text are constructed in and for different (historical or material) contexts, they are not categorically distinct from sociopolitical forms (and I want to add scientific or theoretical forms here). Rather, they are all arrangements of elements, orderings, patternings, or shapings. And precisely because all forms are abstract organising principles, Levine argues, “they are iterable—*portable*. They can be picked up and moved to new contexts” (2015, p. 7 emphasis added). Think of the overture, which has ‘travelled’ from opera to modern literature. Or the binary opposition between subject and object that shape Cartesian thinking,^5^ linguistic typology as well as the Greimasian actantial model.^6^

In the following discussion, I demonstrate how this method informs a study of biological individuality within a broader cultural and social context, taking a broad view of the bounded organism as a paradigmatic traveling form—one that emerges from the interplay of scientific, sociopolitical, and cultural forms, whose overlapping density, to borrow Levine’s words, is “a fact of our most ordinary daily experience” (2015, p. 22). I particularly stress the relevance of narrative fiction for such an exploration, as it not only highlights the biopolitical power of literary forms in co-shaping configurations of individuality like the bounded organism, but also serves as a site where sociopolitical, scientific, and literary forms overlap and intersect, generating potentially unforeseen configurations that may push the biological imagination into new terrains beyond the bounded organism.

### The (bounded) organism: a traveling form

The organism serves as a prime example of how forms of individuality cross contexts. Historically, the term ‘organism’ referred to a specific way of organisation, which later evolved into being adopted as a concept for the fundamental unit of life, as it corresponded with the observations of life’s organisation. This notion of an organism in the life sciences was built at a time, Thomas Pradeu writes, “when evolutionary considerations were scarce, and was used to refer to a unit of functioning, reflecting centuries of thoughts on questions such as part-whole relations” (2016a, p. 10). The biological notion of the organism, in turn, became a template for sociopolitical imaginaries such as the body politic (Latour et al., 2020; see Levine, 1995) and ideological indoctrination, as elaborated on by Victor Klemperer in his comprehensive analysis of the Third Reich’s use of language (1947, p. 156). But the organism served a template for philosophic, aesthetic and poetic contemplations as well, and became a central form of thinking in literary, and particularly formalist theory (Levine, 2015; see Steiner, 2014). “For Aristotle,” Caroline Levine writes, “the work of literature must be ‘whole and complete,’ coming to ‘resemble a living organism in all its unity’” (2015, p. 24). The idea of literary and poetic texts representing an ‘organic’ structure that is more than the sum of its parts, as it has been a central tenet of the work of American New Criticism, for example (*Organic Form*, n.d.), has a long history in Western philosophy, “as do some typical features of organicist approaches, such as wholeness and structural closure” (Gutmann & Neumann-Held, 2000, p. 277). Thus, as they move between dissimilar materials (where differences can be physical, conceptual, or contextual) or systems of discourse (e.g., scientific, poetic, political) “forms bring their limited range of affordances with them. No matter how different their historical and cultural circumstances, that is, bounded enclosures will always exclude, and rhyme will always repeat” (Levine, 2015, p. 7). The organism, understood as a traveling ‘form of order,’ offers a distinctive perspective on part-whole relations, whether applied to animal bodies or societal structures, in a manner different from, say, a swarm or the holobiont. Each of these forms of individuality “lays claim,” to use Levine’s phrasing, “to its own affordances—its own range of capabilities” (2015, p. 10).

However, forms differ, and differing among forms involves seeing how analogy is similar to a game of Chinese whispers—some elements come across, others do not, or they are changed according to need or understanding. As Anne Whiston Spirn writes, “the same material, form, or action may have different meanings in different settings–water in a desert, water in a sea” (2000, p. 11). It is in the literary formalism of American New Criticism, Tilottama Rajan writes, that the organism as metaphor receives its definitive modern restatement. This aesthetic organic form, however, Rajan notes, “conveniently forgets organic ‘process,’ in a supplementation of the organic with structural metaphors (like the well-wrought urn) that is the reverse of Kant’s troubled supplementation of the metaphor of the building with that of the body in his notion of ‘architectonic’ in the first Critique” (2004, p. 46). This is not surprising, given that novels and poems are written by authors (architects), who, with the help of narrative strategies, forms and artistic choices (tools), design worlds and characters (houses) within the confines of language or narrative conventions (similar to the confines of physics). What becomes clear, then, is that the organism as metaphor in formalism conflates with the idea of literature as static buildings, being ‘whole’ and ‘complete’, having ‘structural closure’.

This conflation is not an isolated phenomenon but reflects a broader ‘ontological intuition’ of a world composed of distinct, well-bounded entities (Elwick, 2017; J. Wilson, 1999; Wilson & Barker, 2024). Within this framework, the contents of each entity are unified and exclusive to it, as exemplified by the modern ‘one-genome/one-organism’ doctrine, which emphasises the uniqueness of individual organisms based on their genetic makeup (Gilbert et al., 2012). Such container-thinking, which integrates boundedness and distinctiveness into a unified form of individuality, does not easily align with contemporary science’s emphasis on compositionality, multiplicity, relationality, and, as illustrated with the formalist metaphor, processuality. Nevertheless, its prominence remains resilient as a conceptual habit, tightly woven into cultural and social structures, illustrating how forms of individuality can shape “what it is possible to think, say, and do in a given context” (Levine, 2015, p. 5). As James Elwick notes, “the history of biology shows that the habit of seeing biological individuals as tidy and bounded entities is a difficult one to break” (2017, p. 277). Despite numerous empirical challenges to these preconceptions, briefly discussed in the first section, people consistently revert to traditional definitions. Elwick highlights that the resilience of this ‘bounded organismality’ lies, among other things, in its fit with other sociopolitical or cultural forms that permeate our thinking. These include the previously discussed tendency in Western liberal society to view agency or autonomy as something innate to an individual, rather than emerging from a set of relations (2017, p. 279); the conflation of fundamental change with a sense of identity (2017, p. 278); and the narrative of progress including the ‘great chain of being’ doctrine (2017, p. 282), which categorises life forms through an arboreal, hierarchical structure—from the ‘lower’ or ‘lesser’ ones to the ‘higher’ ones—forming a teleological story. This illustrates how intersecting forms can reinforce one another, necessitating an intersectional analysis that closely examines their compounded effects—a strategic formalism indeed.

### Narrative and the bounded organism

Such a formalist approach to biological individuality can benefit from close attention to narrative fiction in at least two ways. First, literary forms can significantly shape what Elwick refers to as our ‘ontological intuitions’ (2017, p. 277), and, in doing so, carry political power. We are storytelling animals, immersed in a world of symbolic forms (Bruner, 2004; Fisher, 1985; Koschorke, 2018). Works of narrative fiction (formal narrative), therefore, hold particular value as they directly tap into this ‘narrative cognition’ (natural narrative) (Herman, 2017). As Jerome Bruner notes, “The toolkit of any culture is replete not only with a stock of canonical life narratives … but with combinable formal constituents from which its members can construct their own life narratives” (2004, p. 15). To name a trite but telling example, many narrative fictions, particularly the traditional novel, present life as a linear progression through distinct phases, marked by turning points like marriage or social advancement, culminating in a clear resolution. This structure affords a perception of time that leaves little room for simultaneity, multiplicity, or complexity, favouring a linear, cause-and-effect flow. Simple causality is capitalized on in what Abbott (2008) calls the ‘narrative of centralized control’—a playlet that reduces complex emergent behaviour to a distinct agency and significantly influences our interpretation of real-world events, whether in politics or evolution—a narrative inclination that conspiracy thinkers, for example, extend to significant events, assuming they are not emergent from a web of complex relations but part of a hidden, orchestrated plan (Booth, 2025). Such narrative structures have a direct impact on how individuality is configured in fiction, which in turn influences how we make sense of ourselves and other formations of life. As Abbott points out, narrative, because of its need for a narratable thread, is poorly positioned as a tool to grasp emergent behaviour—that complexity of form not predictable from antecedent conditions (2008, p. 228). Since emergence is key to understanding most, if not all, forms of individuality, this already presents a poor outlook. We can narrate, as Abbott points out, a ‘macro level’ and a ‘micro level’—but how one turns into the other (that is, how it forms the ‘whole’) “is lost in a massive distribution of cause among agents” (2008, p. 233). In other words, narrative might feature swarms, but only as a single entity (composed of many) or by focusing on some of its individual parts, losing sight of the swarm they constitute.

To provide another example of how particular literary forms shape notions of individuality, it has been argued that the rise of the modern novel not only coincided with but also significantly shaped the emergence of the modern liberal subject (Armstrong, 2005; Fehrenbacher, 2020; Watt, 1957), whose configuration, already briefly discussed in Sect. 2, is deeply intertwined with conceptualizations of biological individuality. Nancy Armstrong argues that the specific form of the novel—which includes, among other things, novelistic self-narration and particular narrative patterns such as the structural plot that positions a moral subject against socially inculcated systems of value—shapes the very idea of liberal individualism (characterised by qualities such as autonomy, self-possession, and a well-defined and thus well-bounded individuality in relation to society) into a narrative structure, thereby enabling it to take shape and spread throughout the West in the nineteenth century as rapidly and decisively as it did, ruling out other subjectivities that foster a relational or collective mode (2005, p. 10). Key to this dynamic are figures of alterity, which haunt the modern liberal subject as intrinsic antidotes to the ideal of atomistic, bounded individuality; these “extraordinary forms of individuality,” as Armstrong ironically puts it, are “so extravagant that they must be destroyed in the name of humanity … a threat at once to family and nation” (2005, p. 7). Armstrong illustrates this through H. Rider Haggard’s cannibals and Bram Stoker’s vampires, arguing that by merging bodies, these figures reject “the premise that there is one mind per body and one body per mind” (2005, p. 24). Describing them as “phobic representations of the human aggregate” (2005, p. 25), Armstrong illustrates how they shaped the modern subject *in opposition* to a self-consuming otherness or collectivity that threatens bounded individuality. As figures of alterity, they defined the idealized body politic by delineating what it is not, reaffirming the nuclear family and the bounded, discrete nation-state.

To bring the argument into the twentieth century, we can consider an influential work of science fiction like Aldous Huxley’s *Brave New World* (1932), which directly counters the eugenic utopia of J.B.S. Haldane’s *Daedalus* (1924) with a dystopian vision of biological engineering where individuals function as modular units within a larger collective body, shaped by their biological conditioning. Central to Huxley’s dystopian vision is the ‘Bokanovsky Process,’ a fictional cloning method that produces up to ninety-six genetically identical individuals from a single human egg, ensuring uniformity and conformity within the World State. The protagonists respond differently to this ‘socially inculcated system of value,’ with most taking morally antagonistic stances toward the genetically engineered hive society—a “nightmare of swarming, indistinguishable sameness” in the eyes of one of its central antagonists, John, the Savage (Huxley, 1932/1946, p. 250). Drawing on emerging modern synthesis discourse (Clayton 2023), Huxley blends a gene-centred futurism with a phobic vision of collectivist biopolitics, anticipating narratives of genetic selfhood that echo throughout the twentieth century (Creager, 2022).^7^

Modes of human and nonhuman aggregation continue to be central plotting devices throughout twentieth-century science fiction. Works such as *The Colour Out of Space* (1927) by H.P. Lovecraft, *Invasion of the Body Snatchers* (1956) by Jack Finney, and *The Puppet Masters* (1951) by Robert A. Heinlein explore fears of losing self—biologically, as control over one’s body and mind is ceded; socio-politically, as individuals risk succumbing to the parasitic influence of a collective or ‘more-than-self,’ or through the proverbial parasitic invasion of outside elements into the body politic. The figure of the parasite, in fact, serves throughout the twentieth century as an emblem of societal and biological anxieties about collective life, relationality, and (inter)dependence; and much like the organism, it exemplifies how forms of life cross materials and discourses throughout history, carrying with it the affordance of a particular body politic defined by boundedness, where the (national) organism under attack is perceived as distinct from an environment teeming with potential threats (Kaishian et al., 2022; Musolff, 2014, 2016; Sapp, 1994; Waśniewska, 2017). Emerging from the Germ Theory paradigm as a symbol of (biological) invasion and contagion, it introduces a particular order to relationships when close associations between different entities emerge, positioning distinct entities in a survival-of-the-fittest dynamic. In this context, one feeds off another to its detriment, creating a tension between exploitation and dependency, whether we consider a tapeworm and its human host or the dependence of the wealthy elite on the labour of the working class—a recurrent analogy that has gained renewed traction in Bong Joon-ho’s film *Parasite* (2019). On one hand, through metaphors of attack, invasion, and defence, the parasite responded to and naturalized the anxieties of an era marked by conflicts between nation-states and societal ideologies. On the other hand, as a phobic representation of aggregation, it reinforced “the ‘natural’ order of otherwise discrete and independent individuals” (Kaishian et al., 2022, p. 7).

What becomes clear from this discussion is that the bounded organism emerges from the overlapping density of scientific, sociopolitical, and cultural forms, each contributing to an ‘ontological intuition’ of individuals as discrete, autonomous, well-bounded entities. The assumption of its ‘naturalness’ structures both bodies and body politics. Turning to narrative fiction means recognizing that the stories we tell in specific historical contexts draw from their respective biological discussions about form—whether concerning parasitism, biological aggregation, or (a-)sexual reproduction—using these forms as plotting devices and integrating them into narrative structure, which, in turn, has significant consequences for how we think not only about the self and politics but also about the structures of life that inspired these stories in the first place.

### Narrative beyond the bounded organism

However, there is another way in which a closer look at narrative fiction can be productive—one that challenges the causal reading outlined above. Works of narrative fiction are often not univocal in their ideological anticipation, but rather sites of ambivalence, conflict, and negotiation. The sharp edges of Aldous Huxley’s *Brave New World*, for example, cut in many directions. While it undeniably offers a critique of the scientism of its time, the novel’s plot does not revolve around a simple moral conflict (Clayton, 2016). Instead, it works out a multiplicity of overlapping and interacting forms—from Fordist assembly lines and ectogenesis to political totalitarianism, Shakespearean drama, and overly romantic individualism—its overlapping density contributing to the satirical whims of the narrative. Likewise, as the long history of literary formalist experimentation shows, narrative fiction can productively break narrative expectations, and at times, it must. To effectively engage the reader, for example, it is crucial to disrupt repetitive patterns and defamiliarize the familiar—a principle of narrative fiction (and of art in general) first articulated by Shklovsky and later expanded across various media by others (Lippert, 2024). When it comes to defamiliarizing the ‘imaginary essences’ by which we individualise, to use Elwick’s phrasing (2017, p. 277), literary fiction can serve as a site of experimental subversion. Here, normative forms of individuality are potentially estranged through their conjunction with defamiliarizing techniques (omission, juxtaposition, etc.) and/or with strange forms of individuality that, unlike the ‘phobic representations of the human aggregate’ discussed above, do not act as norm-affirming monstrosities but rather as mirrors—sites of reflective ‘weirding’ that unsettle what is taken as natural.

Works of science fiction occupy a unique position in this regard, as the genre both explicitly engages with scientific knowledge and knowledge-making practices and fundamentally operates through the mode of estrangement as famously typified by Darko Suvin (1979, see also Bayraktar & Godioli, 2024, p. 3). To illustrate this with some notable examples, Stanislaw Lem’s *Solaris* (1961) explores the limits of human understanding and communication when encountering an alien intelligence whose biological nature defies conventional scientific categories of delineation and individualisation.^8^ Frank Schätzing’s *The Swarm* (2004) reimagines Lem’s *Solaris* in the context of present-day ecological disruption, where a group of scientists strives to understand a microbial, swarm-like intelligence emerging from the ocean depths in a ‘nature strikes back’ narrative. The scientists are particularly challenged by the creature’s dual nature, as both a collective of individuals and a singular entity of multitudes, prompting a reflection on human biological individuality as well (Brandt, 2025; Dürbeck, 2012). Octavia Butler’s *Xenogenesis Trilogy* (1987–1989) grapples with the consequences of human-alien symbiogenesis, weirding biological essences through interspecies fusion, and allegorising the story of our ‘alien’ mitochondria, thus directing our attention directly from the ‘anomalous’ to the image of ourselves (Bollinger, 2010; Clarke, 2008). Jeff VanderMeer’s *Southern Reach Trilogy* (2014–2015), in turn, presents a mysterious ecological phenomenon known as Area X, which constantly shifts and defies scientific explanations, unsettling our assumptions about the natural world and our place within it (Ulstein, 2017).

Fictional narratives are particularly valuable for a strategic formalism because, as Levine writes, they “allow us to imagine the subtle unfolding activity of multiple social forms” (2015, p. 19). A closer look at narrative fiction, then, not only implies excavating subtle textual interventions to normative or intuitive models of individuality but also tracing how diverse forms unfold and how their collisions can unexpectedly drive social change. Science fiction, in particular, is where scientific theory intersects with social structures, cultural imagination, and speculative futures; where individuals are formed through the overlapping density of multiple signifying institutions (science, politics, technology, law, etc.), along with the more fundamental structures of the narrative itself. In light of the works mentioned above and the previously discussed argument that the form of the novel is deeply intertwined with a biopolitics of bounded organismality, then, it becomes essential to analyse, first, how anomalies to this model are narrated, the formal strategies used to represent them, and their implications for our understanding of literary and poetic forms; on the other hand, to explore what ‘anomalous’ forms afford beyond their source domain of biological practice and theory. Weird forms of life might reveal unexpected collisions or amplifications with the forms that permeate contemporary culture. We might ask, for example, how figurative holobionts interact with narrative structure (Brandt, 2023) or what happens when complex agential structures—such as those pertinent to swarms—meet with simple narrative causation (Brandt, 2025). I use the term ‘weird’ because such analytical practice involves thinking beyond the human body as the ‘fulcrum of proportions’ (Foucault, 2001, p. 26) and, much like what Mari Bradic has summarised as the practice of ‘weird science’, significantly reorients our sense of reality and our place within it (2020). This also involves thinking about how literary forms afford thinking beyond conventional (read: meso-, human-sensory focused) scales. Marco Caracciolo investigates, for example, the formal configurations through which narrative can channel our involvement in nonhuman processes, including the profound, planet-scale disturbances such as climate change (2021). In a similar vein, Liliane Campos theorises the disruptive epistemic work performed by the topos of the microcosm, comparing its analogical and synecdochical forms (2023). In light of this ‘new formalist’ awareness, we might revisit the noncontemporary as well, as exemplified by a recent volume edited by Alberto Godioli and Carmen van den Bergh that explores how modernist texts blur the borders between different ‘forms of life’ through literary experimentation, as “the formal innovations brought about by modernism can serve as an invaluable inspiration for the posthumanist imagination of the twenty-first century” (2023, p. 10).

My rough flight through possible trajectories of a formalist approach aims to demonstrate that forms *matter*. For forms of individuality, this significance emerges from the political work they do through the analogical reasoning they afford. For sociopolitical, literary, and poetic forms, it lies in how these forms intersect, clash, and anticipate forms of individuality, either enhancing or subverting them. In both cases, forms function as abstract organising principles, operating intuitively as ‘ontological intuitions’ or theoretically as scientific forms of individuality, colliding with other forms through which we read and disclose worlds—whether real or fictional—such as narrative structure, prominent tropes, and literary devices. However, formal analogies of life never exist as mere abstract artifacts; instead, they always emerge within a dialectical relationship with material conditions. Matter *forms* and forms *materialise*—a dynamic that requires further discussion, particularly if we aim to understand how and why we individualise as we do.

## Forms (and) matter

Levine writes that “a reading practice that follows the affordances of both literary forms and material objects imagines these as mutually shaping potentialities, but does not fold one into the other, as if the materiality of the extratextual world were the ultimate determinant” (2015, p. 10). On the contrary: patterns and arrangements shape matter, “imposing order on stone and flesh, sounds and spaces … things take forms, and forms organise things” (2015, p. 9). Thus, for Levine, there is a materiality of discourses and a semiotics of materials—and form takes centre stage. Although Levine does not explicitly frame her argument in these terms, her approach suggests a dialectical understanding of form and material reality, in line with the tradition of Marxist formalist thought, in which cultural objects are social acts that transform and are transformed by the material conditions from which they arise (cf. Eagleton, 1976; Jameson, 1971/2016, 1982).^9^ To recall Eagleton’s ‘double-edged relationship’ (see Sect. 2), form is not a passive mediator but actively shapes material conditions, while material conditions, rather than merely awaiting discursive articulation, exert their own affordances. Calling this dynamic ‘dialectical’ highlights the reciprocal, generative tension between meaning-making and material conditions, with neither fully determining the other.^10^ This aligns with a prevalent tendency in critical theory to extend the concept of semiosis—that is, the process of creating meaning through signs—beyond the confines of the (literary) text or even beyond language, assuming what Donna Haraway referred to as a material-semiotic reality (2003, p. 2). In their introduction to *Material Ecocriticism*, Serenella Iovino and Serpil Opperman write that, for the recent group of scholars united under the moniker of New Materialism, “reality emerges as an intertwined flux of material and discursive forces, rather than as a complex of hierarchically organized individual entities” (2014, p. 3).^11^ From this standpoint, integrating a new materialist perspective proves crucial: *the structures through which we individualise afford specific bodily experiences*, particularly how we perceive ourselves to be situated in and against an environment. Likewise, forms of individuality afford distinct ways of assembling the natural world, directly shaping how life and matter are organized and materialised. As new materialist theorist and physicist Karen Barad (2007) writes, “the world is materialized differently through different practices” (p. 88). Conversely, how we experience our bodily being-in-the-world also affords particular ways of thinking about individuation. As Levine observes, “some impositions of order are discursive, others are embodied” (2006, p. 635). In regard to forms of individuality, they are often both discursive and embodied; they are, as McConwell puts it, “complex junctures of bio-social spaces” (2023, p. 57).

One prominent example of how certain forms of life shape matter is the prophylactic use of antibiotics, which is rooted in the historical vicissitudes of taxonomy, where bacteria have long occupied an abject status (Hird, 2009). This intuitive modeling has significantly altered microbiome composition—not only in humans and domesticated animals, but also in ecosystems at various trophic levels through antibiotic residues (Grenni et al., 2018; Polianciuc et al., 2020). “This biological matter,” Hannah Landecker writes, “chewing away its own ontology, is historically and culturally—and materially—specific to late industrialism, produced in and by previous modes of knowledge” (2016, p. 21). Another example is how forms of individuality inform and shape perceptions and decisions regarding childbirth. For example, we might ask how certain narratives of individuality inform particular practices during childbirth, in line with Scott F. Gilbert’s call for a re-telling of the human birth narrative in light of holobiont research (2014). The deterministic narrative of genetic selfhood as articulated in Huxley’s *Brave New World*, for example, continues to resonate as a rhetorical commonplace in contemporary IVF discourse (Lippe, 2017; Lynch, 2019; McQuillan, 2006). Or we might examine the affordances of different conceptual frameworks in determining when, or if at all, an organism ‘begins’ (Kingma, 2018), and what this tells us concerning, for example, abortion politics (Takeshita, 2017). Again, the intuitive modelling of entities *as containers* is relevant here and is problematized (Kingma, 2019). On a whole different level, forms of individuality afford conservation strategies, for example through moving away from a focus on ‘charismatic megafauna’ to taking into account the larger multispecies assemblages that are essential for the functioning of a particular endangered species (Carthey et al., 2020). In short, forms of individuality not only shape particular ways of thinking and knowing, but also actively participate in assembling the ‘natural,’ which has real socio-political consequences*.*

Conversely, the human body, as experienced, serves as a powerful reservoir for models of visibility. Foucault highlights that the space of analogy is one of radiation, with the human body acting as the focal point where relationships are both concentrated and reflected back into the world (2001, p. 26). In accordance, Scott Atran notes that life forms have historically been evaluated against human morphological and behavioral standards, often relegating those lacking ‘phenomenal resolution’ to residual categories (1993). In this sense, *every politic is a body politic*. To illustrate, in *Metaphors We Live By*, George Lakoff and Mark Johnson argue that, at the outset, “we are physical beings, bounded and set off from the rest of the world by the surface of our skins, and we experience the rest of the world as outside us. Each of us is a container, with a bounding surface and an in–out orientation” (1980, p. 29). They argue that we project this embodied experience of containment onto the world as a ‘metaphor we live by’: we “impose boundaries—marking off territory so that it has an inside and a bounding surface—whether a wall, a fence, or an abstract line or plane,” transforming solid objects into ones that have an inside or demarcating fuzzy areas, such as the ‘border’ of a wood clearing (1980, p. 29). Lakoff and Johnson thus root container-thinking in our direct, embodied experience. But does that mean that the way we individualise is bound to be bounded, as Lakoff and Johnson suggest? Is bounded organismality ingrained in human cognition, perhaps because it is cognitively economical and therefore intuitively preferred, as Elwick (2017, p. 279) proposes? And are we, therefore, stuck with an analogical radiation of containment, able to oppose it only through careful empirical and theoretical scientific knowledge-making? To address these questions, we must consider how ‘anomalous’ conceptions of biological individuality—such as composite organisms, superorganisms, holobionts—reverberate not only back onto material bodies but also into lived social experience.

As Tobias Skiveren thoroughly identifies, new materialist thought emphasises fictions, or ‘proxy stories,’ as epistemological tools “to move beyond anthropocentric regimes of truth” (2022, p. 189). While science may convince me of the existence of myriad microbial companions essential to my body’s functioning—thus promoting a holobiontic perspective of selfhood—I still find it challenging to internalise or relate to this perspective. Turning to fictionality, then, becomes a way to capture the intangible through affective means, dramatising the scientific ‘weird’ by employing defamiliarisation techniques. These include narrative forms that destabilise ‘intuitive ontologies,’ such as anthropomorphism, zoomorphism, shifts in focalization, world-building, and alternation between micro- and meso-scales. In this sense, as Skiveren puts it, literature cultivates “more matter-attuned and fine-grained sensibilities. Rather than positing an epistemological aporia of matter (instead of signs), several key figures of New Materialist thought view literature as a privileged site for affectively and imaginatively exploring the world of material forces” (2018). In a new materialist framework, narrative fictions thus possess ‘material agency’ because their ordering is inherently constitutive: through the arrangement of events and information in a narrative, narratives shape bodily assemblages. This, once again, highlights the need for a close reading of literary forms and forms of individuality in studying their affordances to bodies of knowledge.

In this spirit, I turn to the holobiont as a more suitable body politic for illuminating the ‘body’ of literary works, rather than relying on the structuralist metaphor of the (bounded) organism. I draw on Scott F. Gilbert’s perspective, who writes that:

We can represent art and biology as parts of a holobiont organism. Looking at it one way, art and biology constitute a single composite artscience organism. Looking at it another way, art and biology are distinct entities that can interact to form new types of structures. This dual Gestalt of foregrounding/backgrounding alternations between single and composite units characterizes physical biological bodies. It may also characterize bodies of knowledge. (2024, p. xx)

The holobiont form, as a composite entity, reflects the dialectical principle of totality—that to understand a phenomenon fully, one must consider the interplay of parts and the whole. To speak with Levins and Lewontin, “part *makes* whole, and whole *makes* part” (1985, p. 272). In this way, the holobiont affords not only a specific epistemological or methodological perspective, but also an ethical stance. As Donna Haraway puts it, particular ‘science art worldings’ are holobiomes, “in which scientists, artists, ordinary members of communities, and nonhuman beings become enfolded in each other’s projects, in each other’s lives” (2016, p. 71), urging us to recognize the mutual responsibilities and shared investments in the world-making processes we engage in. Taking the holobiont metaphor further, its symbionts can either be parasites or mutualists. In other words, they can be detrimental or productive for a particular body of knowledge. However, with holobiont symbiosis, there are no inherently bad or good microbes; everything depends on the context (Méthot & Alizon, 2014; Overstreet & Lotz, 2016). In closely reading different forms of order as they operate in the world—the thinking they afford, the interactions they cause—we might, then, like a holobiont researcher, shift between different scales, moving from the metaphorical microscope to the broader perspective of the artscience Gestalt in an alternating manner, from the multitudes of individuals to the individual of multitudes, and back again.^12^

## Conclusion

Whether through scientific practices like holobiont research, the embodied experience of individuality, biopolitical configurations of childbirth, or narrative defamiliarization techniques in literature, forms operate across various scales, assembling social, natural, and fictional worlds. From this vantage point, the outlined formalist method critically engages with specific conceptualizations of biological individuality *as forms*—orderings, patternings, or shapings through which we interpret and construct worlds, whether they are fictional or real. By situating phenomenal, cultural, and theoretical forms of individuality—though epistemologically distinct—on the same plane, this approach fosters a dialogue that bridges debates on biological individuality in biology and philosophy with formalist reading practices in literary, cultural, and sociological studies, deviating from the premise that forms order, and therefore, are inherently political. Narrative fiction, in this regard, emerges not only as a site where formal configurations radiate from and anticipate sociopolitical discourse but also as a particularly powerful site of convergence, where multiple forms—literary, sociopolitical, and scientific—unfold, collide, and, in their dramatic and affective interactions, create unexpected assemblages that may speak to bodies, which, in turn, may be affected, morphed, and re-imagined in ways *beyond the bounded organism*. If Félix Martínez-Bonati tells us that the “fundamental compass of narrative is a world of individuals,” and Scott F. Gilbert, Jan Sapp, and Alfred I. Tauber dramatically proclaim that “we have never been individuals” (Gilbert et al., 2012), then it seems our narrative compass might need a little formal recalibration.

## Footnotes

1 Throughout this paper, I avoid using the distinction between epistemic and non-epistemic values, unlike McConwell (2023), for example. While this conceptualization is undoubtedly valuable within its respective context, it is somewhat limiting in a discussion of traveling forms, which emphasizes—or even emerges from—the insight into the mutual contingency of epistemic and non-epistemic domains. Further exploration of this distinction falls outside the scope of the paper.

2 This is a general definition and does not encompass the wide range of kinds of biological individuals that have been proposed. For example, genes or lineages, while not typically considered living entities, can still be regarded as biological—and particularly *evolutionary*—individuals, as some have proposed (Dawkins, 1976; Hull, 1976; see also Godfrey-Smith, 2009). The subsequent discussion outlining some problems of biological individuality is far from exhaustive of the broader debate and should be seen only as an introduction to the relevance of formalist thinking in this context. For a comprehensive introduction to the problem of biological individuality, see, for example, Wilson and Barker (2024) or McConwell (2023).

3 While Levine acknowledges both Jacques Rancière and Michel Foucault (Levine, 2006, p. 75, 2015, p. 3), Rancière’s political theory draws significantly from Foucault’s ideas. Thus, we might trace the concept of politics as administration back to Foucault (see May, 2008).

4 See Wehrle (2016) for an extensive discussion of how, according to Foucault, practices of discipline become ‘embodied’ and how this relates to a phenomenological perspective.

5 For discussion of how Cartesian rationalism is embedded in cultural and historical shifts, see Susan Bordo’s *The Flight to Objectivity* (1987).

6 The Greimasian actantial model structures narratives through the subject/object dynamic, where the subject strives to attain or act upon an object.

7 Huxley further explores his socio-genetic ideas in *Brave New World Revisited* (1958), where he advocates for “individual freedom, based on human diversity and genetic uniqueness,” laying out his gene-centred body politic (Huxley, 1958/1974, p. 149). Genetic determinism, as emblematic of Huxley’s vision, has been widely criticized (e.g., Keller, 2009; Lewontin et al., 1985), but persists in public discourse to this day (Moore, 2008). Maurizio Esposito attributes this persistence to the ‘rhetoric of futurity,’ where imagined worlds shape “the framework in which technologies and ideas are shaped and thrive,” underlining the epistemic work of narrative speculation such as Huxley’s (2017, p. 8).

8 Lem’s famous novel has inspired a vast array of interpretations, and arguably, this ‘totality of interpretations’ mirrors the difficulty of interpreting the biological anomaly it portrays. For a recent perspective, see Slawkowska-Rode & Slawkowski-Rode (2021).

9 Levine might object, given her skepticism toward simplistic dialectical analyses that reduce complex interactions to oppositions, advocating instead for “contingent formal encounters” where different forms “are not necessarily related, opposed, or deeply expressive” (2015, p. 18). While this is a valid caution against reductive thinking, I believe that dialectics, as a critical tool, remains indispensable and does not preclude the possibility of other modes of thought—which has been made particularly evident in the context of environmental concerns (Foster et al., 2010; Moore, 2015).

10 Thinking dialectically in this way reflects a long-standing tradition in biology as well that examines the entanglement of scientific, social, and ideological factors through a Marxist dialectical materialist lens—a view central to the work of Levins and Lewontin (1985, 2007). See also Foster (2020).

11 There is considerable debate over the relationship between Marxist dialectical materialism and New Materialism, particularly regarding whether the latter is genuinely ‘new’ or simply a departure from—or dismissal of—its Marxist predecessors without sufficient justification (cf. Newman, 2022a; b; Skiveren, 2023). While this debate lies beyond the scope of this article, in short, I see no inherent contradiction between these two ‘schools of thought’ per se (neither of which is monolithic) and, in the context of the discussion at hand, much more in their points of convergence, as the holobiont metaphor at the end of this section aims to show.

12 I have used this metaphor previously in (Brandt, 2023).
